# 

**Published:** 1891-08-22

**Authors:** 


PRICE ONE PENNY.

No. 256, Vol. X.—August 22nd, 1891.

[Registered at the General Post Office as a Newspaper for transmission in the United Kingdom and Abroad.]

WITH EXTRA NURSING SUPPLEMENT.

Per Annum, 6s. 6d.; post free.

Monthly Parts, 6d.; post free 8d.

Ditto per Annum, 8s., post free.

### THE HOSPITAL

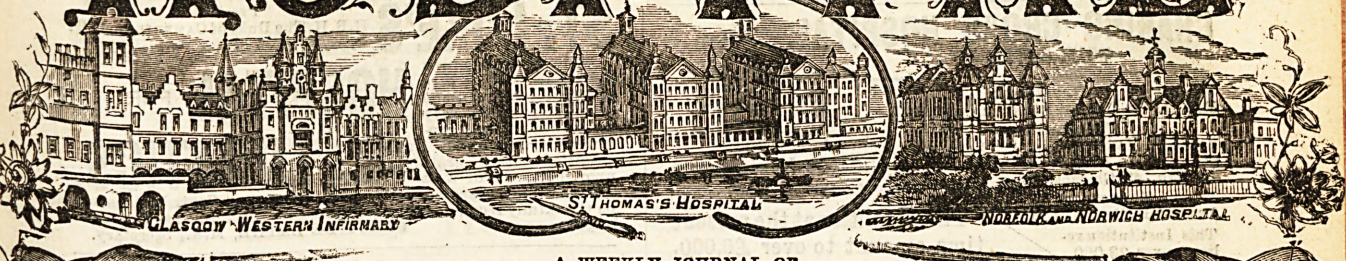

A WEEKLY JOURNAL OF

### Science, Medicine, Nursing and Philanthropy.

SATURDAY, AUGUST 22nd, 1891.

**Municipalities and Public Health** - 241

**Passing Topics.**—Rooks and Retribution - 242

**More than Brothers** - 243

**Employments for Women.**—III.—Gardening - 244

**Everybody's Page** - 245

**Notes and News** - 246

**General Charities** - 247

**Scraps and Gleanings** - 247

**Annotations.**—Out of the World—Nurses in Workhouses—The Cause of Tetanus - 248

**Lady Strangford Hospital, Port Said** - 252

**The Practitioner's Mirror and Retrospect—**

MEDICINE.—Gastrectasis (*continued*). B—Diagnosis - 249

SURGERY.—Diseases of the Fingers: C—Contractions (*continued*) - 249

THERAPEUTICS.—On the Use of Creosote in Phthisis, 250; On the Action of Salicylates in Certain Rheumatic Diseases - 251

**The Story of the Insane from Year to Year** (Fourth Series) - 252

**The Student of Medicine.**—Appointments—Vacancies—Pass Lists

EXTRA SUPPLEMENT—THE NURSING MIRROR.

**En Passant.**—Weymouth Royal Hospital—Sheffield Nurses' Home—Nurses and Doctors—Hospital Nurses—Queen Victoria's Jubilee Institute for Nurses - cxix

**Lectures on Surgical Ward Work and Nursing** By ALEXR. MILES, M.B.Edin., C.M., F.R.C.S.E. XXXIII.—Bandages for the Head - cxx

**Christmas Competitions** - cxx

**For Reading to the Sick**—Contentment.—II. - cxxi

**A Uniform Chat** - cxxi

**The Royal National Pension Fund for Nurses** - cxxii

**The Princess of Wales' Fund for Mrs. Grimwood** - cxxii

**The Nurses' Bookshelf.**—A Series of Text-Books—Betwixt Two Lovers - cxxii

**Derby Children's Hospital** - cxxiii

**The Princess of Wales and the Nurses** - cxxiii

**Appointments** - cxxiii

**Notes and Queries** - cxxiii

**Off Duty.**—The Other Benny - cxxiv

**Wants and Workers** - cxxiv

**Amusements and Relaxation** - cxxiv

